# Fever and Antipyretic Supported by Traditional Chinese Medicine: A Multi-Pathway Regulation

**DOI:** 10.3389/fphar.2021.583279

**Published:** 2021-03-22

**Authors:** Le-Le Ma, Hui-Min Liu, Chuan-Hong Luo, Ya-Nan He, Fang Wang, Hao-Zhou Huang, Li Han, Ming Yang, Run-Chun Xu, Ding-Kun Zhang

**Affiliations:** ^1^ State Key Laboratory of Southwestern Chinese Medicine Resources, Pharmacy School, Chengdu University of Traditional Chinese Medicine, Chengdu, PR China; ^2^ State key Laboratory of Innovation Medicine and High Efficiency and Energy Saving Pharmaceutical Equipment, Jiangxi University of Traditional Chinese Medicine, Nanchang, PR China

**Keywords:** COVID-19, fever, antipyretic, traditional chinese medicine, mechanism, bioactive components

## Abstract

The coronavirus disease, 2019 (COVID-19), has spread rapidly around the world and become a major public health problem facing the world. Traditional Chinese medicine (TCM) has been fully committed to treat COVID-19 in China. It improved the clinical symptoms of patients and reduced the mortality rate. In light of the fever was identified as one of leading clinical features of COVID-19, this paper will first analyze the material basis of fever, including pyrogenic cytokines and a variety of the mediators of fever. Then the humoral and neural pathways of fever signal transmission will be described. The scattered evidences about fever recorded in recent years are connected in series. On this basis, the understanding of fever is further deepened from the aspects of pathology and physiology. Finally, combining with the chemical composition and pharmacological action of available TCM, we analyzed the mechanisms of TCMs to play the antipyretic effect through multiple ways. So as to further provide the basis for the research of antipyretic compound preparations of TCMs and explore the potential medicines for the prevention and treatment of COVID-19.

## Introduction

COVID-19 has become a major threat to worldwide public health, having rapidly spread to more than 180 countries and infecting over 1.6 billion people. Fever, cough, diarrhea, and fatigue are the most common initial symptoms of COVID-19 (Luo et al., 2020a). However, fever was identified as leading clinical feature. A study of the clinical progression of COVID-19 patients in Shanghai, China, included 249 confirmed cases of COVID-19 from Jan 20 to Feb 6, 2020. The research found that as high as 94.3% of the patients, including those who were afebrile on admission had fever. The estimated median duration of fever in all the patients with fever was 10 days after onset of symptoms. Patients who were transferred to ICU had significantly longer duration of fever as compared to those who were stable, up to 31 days (Chen et al., 2020). Clinical data from another study showed that only 43.8% of patients presented with a fever, but 88.7% developed a fever after hospitalization, indicating the afebrile patients may be at the early stage of the disease (Guan et al., 2020). Therefore, preventive treatment should be carried out for a large number of suspected cases and their close contacts in order to reduce the possibility of infection and block the spread of COVID-19. In addition, timely treatment of confirmed patients can prevent further deterioration of the disease, reduce the chance of patients with mild symptoms becoming serious. In light of fever was the most common initial symptom in patients with COVID-19, it is of great significance to understand the mechanisms of fever and take antipyretic measures for diagnosis, treatment and prognosis of COVID-19 patients.

Currently, the treatment of fever includes physical cooling and antipyretic medications such as NSAIDs and Paracetamol. Research has shown that patients with moderate fever should avoid active cooling because it increases the metabolic rate, activates the autonomic nervous system, and provokes thermal discomfort (Lenhardt et al., 1999). In addition, on March 16th^,^ 2020, the French Minister of Health has announced that NSAIDs may worsen clinical conditions of patients with COVID-19 based on the evaluation of four patients affected by the disease (Capuano et al., 2020). Some authors have suggested that NSAIDs, particularly Ibuprofen, may induce increased sensitivity to more severe clinical features in COVID-19 infection (Laura et al., 2020). They argued that coronaviruses bind to angiotensin-converting enzyme-2 (ACE-2), and ibuprofen administration can increase the bioavailability of ACE-2, thus potentiating and enhancing the infectious processes of coronaviruses (Fang et al., 2020). Since nowadays no scientific evidence establishes a correlation between NSAIDS and the worsening of COVID-19, patients should be advised against NSAIDs when COVID-19 like symptoms begins (Fang et al., 2020). Although it has been suggested that patients could take paracetamol to treat the symptoms of COVID-19, overdose or long-term use of Paracetamol can also produce the largely irreversible hepatotoxicity and incipient gastric toxicity (Whitehouse and Butters, 2014). Therefore, it is essential to explore a safe, effective, and low toxicity treatment method. TCM has attracted global attention due to its low toxicity and high efficacy. According to clinical observations, the common symptoms of fever subsided by more than 90% of the 3,698 patients with COVID-19 within 1.74 days on average when the indicated TCM formulation was commenced (Li et al., 2020). TCM has been used to treat fever for more than 2000 years. Many years of clinical observations and several published studies suggest single Chinese medicine and compound preparations of TCM have specific antipyretic effects; these include Bupleuri Radix (Idrisusman et al., 2010), Scutellariae Radix (Tsai et al., 2006), Lonicera Japonica Flo (Xie et al., 2009), Shuang-Huang-Lian injection (Huang et al., 2019), Qingkailing injection (Zhang et al., 2017) and so on.

In this paper, through the systematic analysis of the mechanisms of fever, combined with the chemical composition and pharmacological action of TCM (Table 1), we analyzed the material basis, mechanisms, and characteristics of the antipyretic action of single TCM and its compound preparations to deepen the theory of TCM from the micro-level, and to develop safer and effective antipyretic preparations of TCM, as well as improve the treatment level and promote the rehabilitation of patients with COVID-19.

**TABLE 1:** Descriptive table of the Chinese herbal medicines mentioned in this paper.

Scientific name	English name	Common name	Localchinesename	Parts used
*Bupleurum chinense* DC. and *Bupleurum scorzonerifolium* Willd	Bupleuri radix	Chinese thorowax root	Chai-hu	Root
*Scutellaria baicalensis* Georgi	Scutellariae radix	Baical skullcap root	Huang-qin	Root
Conioselinum anthriscoides ‘Chuanxiong’ (syn. *Ligusticum chuanxiong* Hort)	Chuanxiong rhizome	Rhizoma ligustici wallichii	Chuan-xiong	Root
*Cinnamomum cassia* (L.) J.Presl	Cinnamomi ramulus	Cassia twig	Gui-zhi	Branch
*Forsythia suspensa* (Thunb.) Vahl	Forsythiae Fructus	Forsythia	Liao-qiao	Fruit
*Lonicera japonica* Thunb	Lonicera japonica Flos	Honeysuckle flower	Jin-yin-hua	Flower
*Ephedra sinica* Stapf, *Ephedra intermedia* Schrenk et C. A. Mey and *Ephedra equisetina* Bunge.	Ephedrae Herba	Ephedra erial parts	Ma-huang	Erial parts
	Gypsum Ustum	Gypsum	Shi-gao	
*Pueraria montana var. lobata* (Willd.) Maesen & S.M.Almeida ex Sanjappa & Predeep (syn. *Pueraria lobata* (Willd.) Ohw)	Puerariae Lobatae radix	Kudzu root	Ge-gen	Root
*Bubalus bubalis* Linnaeus	Bubali Cornu	Buffalo horn	Shui-niu-jiao	Horn
*Houttuynia cordata* Thunb	Houttuyniae Herba	Cordate houttuynia	Yu-xing-cao	Herb
*Gardenia jasminoides* J.Ellis	Gardeniae Fructus	Gardenia	Zhi-zi	Fruit
*Andrographis paniculata* (Burm.f.) Nees	Andrographis Herba	Herba andrographitis	Chuan-xin-lian	Herb

## The Definition of Fever

After three revisions, the Commission for Thermal Physiology of the International Union of Physiological Sciences defined fever as the elevation of the set-point of body temperature due to a change in the thermo controller characteristics. It is usually part of the defense response of organism (host) to the invasion of pathogenic or foreign living (microorganism) or inanimate substances. At this level, the core temperature will be maintained for a period of time (Sciences, 2003). In a normal healthy individual, the thermal regulatory network of the body maintains a temperature of 36.2–37.5°C (Prajitha et al., 2019). Many medical and clinical studies regard rectal temperature of ≥38°C or axillary temperature ≥37.5°C as indicative of fever (Shu et al., 2016). It is worth noting that not all temperature increases can be defined as fever. Clinically, there are 2 cases of typical temperature increase: fever and hyperthermia. Contrary to fever, in hyperthermia, the set-point is unchanged; it occurs in response to specific environmental, pharmacologic, or endocrine stimuli. The elevated body temperature that occurs in hyperthermia syndrome can exceed 41.0°C (Niven et al., 2013). Hyperthermia does not respond to typical antipyretics since there are no pyrogenic molecules (Dewitt et al., 2017); this distinguishes fever from hyperthermia.

## The Material Basis of Fever

### Pyrogenic Cytokines

In 1948, Beeson obtained a substance from the granulocytes of the sterile peritoneal exudate of rabbits, which would raise the body temperature of normal rabbits after injection (Beeson, 1948). Subsequently, similar substances were discovered in other febrile animal models, resulting in the release of an elaborated endogenous pyrogen in consequence of the stimulation by exogenous pyrogens (Dinarello, 1999). Howeve, some endogenous substances, such as autoantibody complexes, inflammatory bile acids, may act as pyrogen without exogenous pyrogens induction (Sajadi, 2015). With the acceptance of the term cytokine, the term endogenous pyrogen is no longer appropriate. In order to differentiate cytokines that are intrinsically pyrogenic from those that are not, it is more appropriate to define them as pyrogenic cytokines (Dinarello, 2004).

The pyrogenic cytokine is a part of the autoimmune system. Invasion of a host by exogenous pyrogens triggers a series of immune responses through pathogen-associated molecular patterns (PAMPs), including LPS, lipoarabinomannans, lipoteichoic acid, and viral RNA (Evans et al., 2015; Kumar et al., 2011). PAMPs act through pattern recognition receptors, such as toll-like receptors (TLRs), on immune cells to induce the release of pyrogenic cytokines (Prajitha et al., 2018). The currently recognized major pyrogenic cytokines are interleukin-1 (IL-1), interleukin-6 (IL-6), tumor necrosis factor *α* (TNF-α) (Conti et al., 2004). Systemic injection of LPS in animal models can increase the release of these cytokines into the general circulation (Harré et al., 2002). LPS stimulate TLRs, specifically TLR4, inducing the release of pyrogenic cytokine that induce fever.

#### IL-1

IL-1 is a prototypical inflammatory cytokine for neuroimmune communication. Discovery of the actions of IL-1 in the macrophages, fibroblasts B cells, endothelium, and large granular lymphocytes, showed that IL-1 represents two different molecular forms (IL-1α and IL-1β) and an endogenous IL-1 receptor antagonist (IL-1RA) (Equils et al., 2020). The study demonstrated that IL-1 is essential in the induction of fever as a central injection or intraperitoneal injection of IL-1RA caused significant inhibition of LPS-induced fever (Miller et al., 1997; Smith and Kluger, 1992). In addition, it has been shown that many species can cause fever response to peripheral injection of IL-1α and IL-1β (Dinarello, 1996; Kluger, 1991). The present explanation for this is that IL-1 induces intermediates, prostaglandin E2 (PGE2), and cyclooxygenase-2 (COX-2), which are considered necessary downstream events that mediate peripheral IL-1-induced fever (Ching et al., 2007; Li et al., 2001). IL-1 induced COX-2 and PGE2 depends on the expression of the mitogen-activated protein kinase kinase kinase 7 (MAP3K7) also known as TAK1. It is most likely to induce COX-2 by activating MAPKP38 and c-Jun, which are necessary for fever induction (Ridder et al., 2011). IL-1 activates the inhibitor of nuclear factor B (IκB) kinase 2 (IKK2), which binds to polyubiquitin chains on several upstream molecules, includingTAK1 and TAB3. Activated IKK phosphorylates nuclear factor-κB (NF-κB) inhibitor *α* (IκBα) and then activates NF-κB (Weber et al., 2010). The rapid activation of the NF-κB pathway induced by IL-1 has been proven to be the cause of COX-2 production in cerebrovascular endothelial cells (Nadjar et al., 2005). Therefore, IL-1 can also induce the production of COX-2 and PGE2 by activating the NF-κB pathway.

#### IL-6

IL-6 is produced under a variety of stimuli including tissue damage, viruses, or other proinflammatory cytokines. It is secreted by innate immune cells, as well as endothelial cells, fibroblasts, astrocytes, and epithelial cells (Rincon, 2012). The IL-6 during inflammation and infection is induced via stimulation of cells by IL-1 or TNF-α or through stimulation of TLRSs after binding of PAMPs (Kang et al., 2019). Receptors for IL-6 exist in two forms: a soluble receptor (sIL-6R) and a membrane-bound receptor (IL-6R) (Uciechowski and Dempke, 2020). Studies have shown that IL-6 knockout mice, as well as in animals treated with IL-6 antiserum, produced no febrile response to the peripheral immune response, suggesting that the existence of IL-6 is very important for fever (Nilsberth et al., 2009). IL-6 produced by non-hematopoietic cells is the key component of LPS induced fever; IL-6 produced by hematopoietic cells plays a secondary role in the production of systemic IL-6. However, the phenotype of these cells is unknown and may involve multiple cell types, requiring further study (Hamzic et al., 2013). In recent years, a new study has shown that tissue macrophages are not involved in the early IL-6 response to LPS. CEACAM1, a molecule ubiquitously expressed in the epithelium, neutrophils, activated lymphocytes, negatively regulates the early response of IL-6 to LPS in murine monocytes through the RP105 signaling pathway (Zhang Z. et al., 2019). Therefore, CEACAM1 may be a potential drug target for antipyretic. The present study also sheds light on the issue of the central administration of IL-6 via PGE2 to induce fever (Harden et al., 2008). Recent studies have further confirmed this view that IL-6 binds to IL-6 receptors on brain endothelial cells, and ligand binding induces the expression of the prostaglandin synthase COX-2 through signals involving activator of transcription 3 (STAT3) pathway (Eskilsson et al., 2014).

#### TNF-α

TNF is a cytokine produced naturally by macrophages in response to bacterial infection or other immune sources. According to its source and structure, it can be divided into two types: TNF-α and TNF-β. The former is principally produced by macrophages, T cells, and natural killer cells (Zelová and Hošek, 2013). TNF-α is the first member of cytokine cascade induced by injection of LPS (Roth and Blatteis, 2014). It has been reported that LPS mediated transcription of TNF-α can be divided into two main signaling pathways. The first proceeds through the NF-κB-inducing kinase route, which regulates the phosphorylation of the inhibitory-κB proteins. The second pathway is mediated by the extracellular signal-regulated kinase and MAPKp38 pathways (Haddad and Land, 2002). Peripheral injection of TNF-α in human and experimental animals can rapidly cause fever (Michie et al., 1988; Roth et al., 1998). Intravenous injection of human recombinant TNF (rhTNF) caused fever in rabbits, which also revealed that the pyrogen potential of rhTNF was associated with an increase in PGE2; the mechanism is related to glutathione. The regulation of TNF-α biosynthesis induced by LPS is redox-sensitive and requires the participation of the glutathione mediated signaling pathway (Wrotek et al., 2015). In the presence of glutathione, it can activate the activity of PGE synthase-1 (mPGES-1), to produce PGE2 (Saha et al., 2005; Thorén and Jakobsson, 2000). A number of researchers also reason that TNF-α induces IL-1 *in vivo*; therefore, TNF-α and IL-1 may play a synergistic role in fever production; however, no specific mechanism has been reported.

#### Others

In addition to the typical pyrogenic cytokines described above, some intrinsic cytokines play a role in fever production (Billiau and Matthys, 2009). Studies have shown that intravenous ET-1 can increase the body temperature of rats. In addition, injection of ET-1 into AH/POA also causes fever, indicating that ET-1 is important for fever response (Zampronio et al., 2015). Leptin is an adipocyte-derived hormone that induced the proinflammatory cytokine IL-1β in the brain, resulting in a prostaglandin-dependent fever (Wisse et al., 2004). Substance P (SP) belongs to tachykinin family. When SP is antagonized by peptide SP analogues, fever response induced by LPS for guinea pig and rat is blocked, which indicates the role of SP in fever (Pakai et al., 2018).

### The Mediator of Fever

A standard linkage was shared by nearly all pyrogenic cytokines above: the production of PGE2. Previous studies have shown that COX-2 and PGE2 induction is required for fever (Pecchi et al., 2009). In addition to PGE2, other mediators can also induce the generation of fever. Free radicals, glutamate, and metabolic pathway disorders also play a role in fever.

#### PGE2 in Fever

The febrile response is characterized by an early rapid phase and a delayed late phase (Blatteis et al., 2004). In some experiments, the pyrogenic cytokines were released later, for example, TNF-α was detected 30 min after LPS IV injection, while the level of PGE2 increased rapidly after LPS IV injection, and then increased further 40 min later (Blatteis, 2006; Callery et al., 1991). PGE2 is a lipid-soluble substance that can pass through the blood-brain barrier (BBB), while pyrogenic cytokines are relatively large, lipophobic peptides, and cannot freely pass through the BBB. Therefore, pyrogenic cytokines may not be able to provide rapid fever signals and be involved in maintaining fever (Blatteis et al., 2005). We can speculate that PGE2 is the first to be initiated in the beginning stage of fever, and is the crucial mediator of fever.

Although it seems definite that PGE2 is an essential mediator of fever, it is not certain whether the PGE2 is of the peripheral or central origin. Which stages of febrile pathogenesis are mediated by the peripheral PGE2, and which stages are mediated by the central, these questions require further investigation. The study has shown that the immediate appearance of PGE2 in inferior vena cava plasma after the IV of LPS and puts forward a hypothesis that LPS-activated complement triggers the release of PGE2 by KC (Perlik et al., 2005). The complement component 5a (C5a) is an essential mediator of the febrile response to LPS (Li et al., 2002). C5aR1 is expressed by KC and rapidly activates COX-1-catalyzed PGE2 production (Schieferdecker et al., 1999; Schieferdecker et al., 2001). PGE receptors have four subtypes, EP1, EP2, EP3, and EP4. Studies have shown that EP3 receptors are of great importance in the febrile response. The organum vasculosum of the lamina terminalis (OVLT) and preoptic-anterior hypothalamic area may be the sites where PGE2 acts on EP3 receptors to generate fever (Oka, 2004). In summary, the initial stage of fever may be mediated by peripheral PGE2, and the rapid transmission of fever signals may depend on neural pathways (see below).

PGE2 is also produced by endothelial cells in the brain and released from the arachidonic acid pathway. This pathway is mediated by the enzymes phospholipase A2 (PLA2), COX-2, and mPGES-1 (Blomqvist and Engblom, 2013; Wilhelms et al., 2014). Brain endothelial cells express IL-1 receptor type 1 (Konsman et al., 2004) and TNF-α receptor p55 (Nadeau and Rivest, 1999). IL-6R is normally absent in the brain endothelial cells, but also induced by inflammation. However, even if there is no membrane-bound IL-6R, soluble IL-6R in blood may participate in IL-6 signal transduction through gp130, a constitutively expressed IL-6 receptor signal sensor in endothelial cells (Vallières and Rivest, 1997). COX-2 and mPGES-1 are the target genes of NF-κB and STAT3, and the activation of NF-κB or STAT3 in brain endothelial cells is related to COX-2 and mPGES-1 in these cells (Rummel et al., 2006). Previous studies have stated that pyrogenic cytokines can induce the production of COX-2 and mPGES-1 through various signaling pathways, so the PGE2 produced by brain endothelial cells is primarily mediated by the effect of these pyrogenic cytokines. However, the generation of PGE2 by COX-2/mPGES-1 did not coincide with the fever response (Steiner et al., 2006b). Therefore, although peripheral synthesis of PGE2 may occur in initiating the fever, central synthesis of PGE2 may participate in its maintenance.

#### Oxygen Free Radicals and Glutamate in Fever

The oxygen free radicals include superoxide anion, hydrogen peroxide, and hydroxyl radical. Dose dependent increase of hydroxyl radical level and core temperature in OVLT induced by LPS or glutamate (Huang et al., 2006). It is suggested that the increase of body temperature caused by LPS or glutamate is related to the increase of hydroxyl radicals in OVLT. Pretreatment with hydroxyl radical scavengers significantly reduced the increase of hydroxyl radicals and fever induced by LPS. Glutamate excessively activation of N-methyl-D-aspartate (NMDA) receptor can produce reactive oxygen species (ROS) in the brain (Yang et al., 1996). After pretreatment with NMDA receptor antagonists, the fever and the increase of hydroxyl radicals in OVLT decreased significantly after LPS injection (Hou et al., 2011). In conclusion, LPS or glutamate may cause excessive accumulation of hydroxyl radicals in peripheral blood and CSF, which can be inhibited by hydroxyl radical scavengers or NMDA receptor antagonists. There is evidence that ROS can activate NF-κB as a second messenger, leading to over induction of COX-2 (Oh et al., 2004). After LPS injection, the ROS in the hypothalamus can stimulate the activation of NF-κB and the expression of COX-2, resulting in the excessive production of NO and PGE2, thus causing fever (Huang et al., 2006). The role of oxygen free radicals and glutamate in a fever not only provides a new theoretical basis for people to understand the mechanism of fever but also suggests that anti-free radical injury could be a new way to study the mechanism of fever in the future.

#### Metabolic Disorders in Fever

A febrile response is a systemic pathological process. In recent years, metabonomics has been widely used in the study of febrile response to reveal the pathological mechanism of this system response. Three methods were used to establish fever model in rats, and the characteristics of plasma metabolism in febrile rats were studied: the TCM-induced rats with fever, yeast-induced rats with fever, and 2,4-dinitrophenol-induced rats with fever, to further investigate the common potential biomarkers in rats with fever (Liu et al., 2017b). The changes in plasma metabolites showed that amino acid, fatty acid amides, phospholipid, sphingolipid, fatty acid oxidation, and glycerolipid metabolisms; also, bile acid biosynthesis were related to fever. It has been confirmed that tryptophan metabolism is important in the metabolic disorder in the fever response (Gao et al., 2013b). Tryptophan, an essential amino acid, is the precursor of 5-hydroxy tryptamine (5-HT) (Guo et al., 2014). It was reported that the 5-HT in the hypothalamus was positively correlated with fever induced by yeast (Peindaries and Jacob, 1971). The increased level of tryptophan in the febrile rats leads to the enhancement of febrile response may be due to the increased ability of tryptophan to synthesize 5-HT. *γ*-aminobutyric acid (GABA) and phosphatidylinositol were also increased in the urine of febrile rats (Gao et al., 2013b). The increase of GABA could lead to an increase of temperature through the pathway of Na^+^/Ca2^+^-cAMP in the hypothalamus (Myers et al., 1976; Romei et al., 2012). The increased cAMP can inhibit the phosphoinositide signaling system and further lead to the significant increase of phosphatidylinositol. Therefore, the disorder of tryptophan metabolism in the process of fever can increase the synthesis of 5-HT and cAMP, and further induce the generation of fever.

Metabonomics is widely used in finding new biomarkers of diseases and revealing the potential mechanism of clinical drugs. At present, it is mainly based on the biomarkers of three kinds of biological liquid samples, serum, plasma, and urine, to reflect the changes in body metabolism. However, because the BBB can control the transfer of molecules between the brain and blood, it is suggested that the metabonomic methods of serum, plasma, and urine should be combined with the metabonomics methods of the brain to explore the antipyretic mechanism of TCM. The research results of metabonomics have created a foundation for the treatment of fever by TCM from different perspectives. They indicate whether we can take a nutritional supplement for the imbalance of essential amino acids, fatty acids, phospholipids, and other substances as a new way of fever treatment in the future.

## Transmission Pathway of Fever Signals

The fever activator stimulates the immune cells to produce the pyrogenic cytokines, which are needed to cause fever through PGE2. At the same time, other central media, independent of PGE2, will also act on the thermoregulatory center to cause fever. At present, with the study of these fever signals and their role when entering the center, it was concluded that the fever response was ultimately regulated in two ways: the humoral and neural pathways (Zeisberger, 1999).

### The Humoral Transmission Pathway of Fever Signals

**FIGURE 1:**
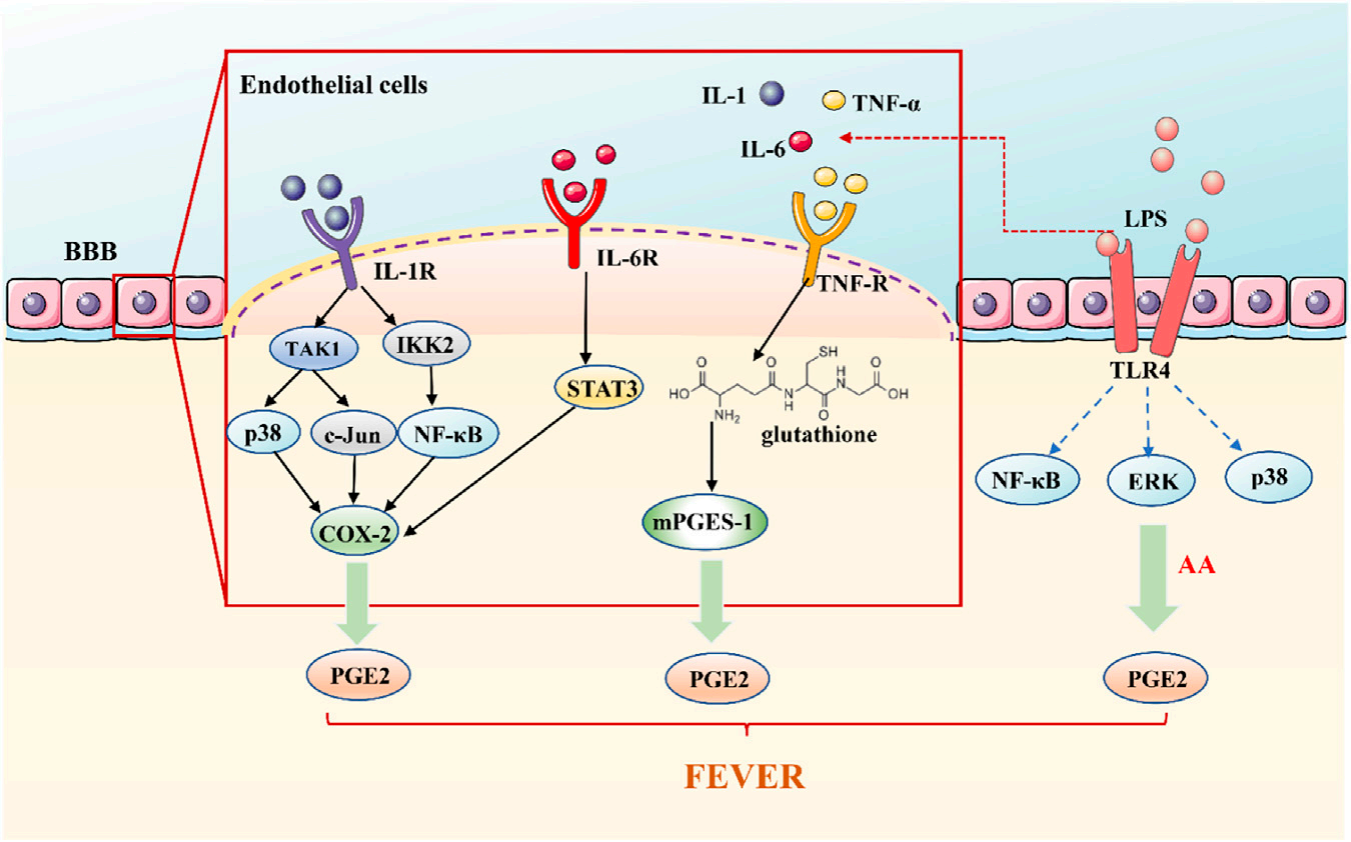
The Humoral Transmission Pathway of Fever Signals. Circulating PAMPs, represented by LPS bind to TLR-4 on the fenestrated capillaries in the BBB. Triggering TLR-4 induces the transcription of COX-2 to converted into PGE2, causing fever; pyrogenic cytokines, TNF-α, IL-1, and IL-6, play a role outside the brain by activating cytokine receptors located on the CVO, resulting in the release of PGE2 to cause fever.

A classical theory of fever signal transmission is the theory of the humoral transmission pathway. Fever signals are carried by PAMPs or by pyrogenic cytokines in this pathway (Figure 1) (Ogoina, 2011). It is known that circulating PAMPs represented by LPS bind to TLR-4, which is located in the fenestrated capillaries of the circum ventricular organ in the BBB. Triggering TLR-4 induces the transcription of COX-2 via NF-κB, MAPKp38, and extracellular signal-regulated kinases (ERK1/2).Also cPLA2 phosphorylation and arachidonic acid mobilization, via MAPK or Mitogen- and stress-activated kinases (MSK1), which is, in turn, converted into PGE2 by COX-2 and released into the cell membrane (Salvi et al., 2016); which through the BBB, then activate the thermal neurons in the front of the hypothalamus, causing fever (Steiner et al., 2006a; Turrin and Rivest, 2004).

According to the classical concept, exogenous pyrogens stimulate peripheral mononuclear phagocytes to produce pyrogenic cytokines, principally TNF-α, IL-1, and IL-6, which are transported from the blood to the brain (Blatteis, 2006; Blatteis et al., 1998). Pyrogenic cytokines are unable to enter the center to stimulate the brain structures through the BBB (Rothwell et al., 1996). At present, there are several opinions on how pyrogenic cytokines are involved in the brain. Pyrogenic cytokines may directly penetrate the sensory circumventricular organs (CVOs) without BBB. Pyrogenic cytokines bind to and activate cytokines receptors on the fenestrated capillaries of the CVO which act outside the brain, leading to the release of PGE2 (Conti et al., 2004; Ogoina, 2011). Vascular endothelial cells and perivascular cells within the entire brain, have been shown to secrete IL-6, PGE2, and other mediators into the brain parenchyma after being stimulated by inflammation in the lumen (blood) side (Blatteis et al., 2000; Roth and Blatteis, 2014; Schiltz and Sawchenko, 2003). In the humoral pathway of fever signal transmission, pyrogenic cytokines can enter the brain directly through the tissue lacking BBB to cause fever or to release PGE2 outside the brain indirectly or stimulate the BBB cells to deliver fever medium to the brain's endocrine system and cause fever.

Due to initial detection of pyrogenic cytokines in the blood does not coincide with the fever caused by IV LPS, the hypothesis of the humoral pathway is not enough to explain the whole process of fever signal transmission. Therefore, an accelerated pathway of fever signal transmission, the neural pathway, has been proposed. The neural pathway, as a supplementary pathway of the humoral pathway, functions in the conduction of fever signals in coordination with the humoral pathway. The conduction process of the two pathways is complex, so the mechanism of their synergistic effect requires further study.

### The Neural Transmission Pathway of Fever Signals

**FIGURE 2:**
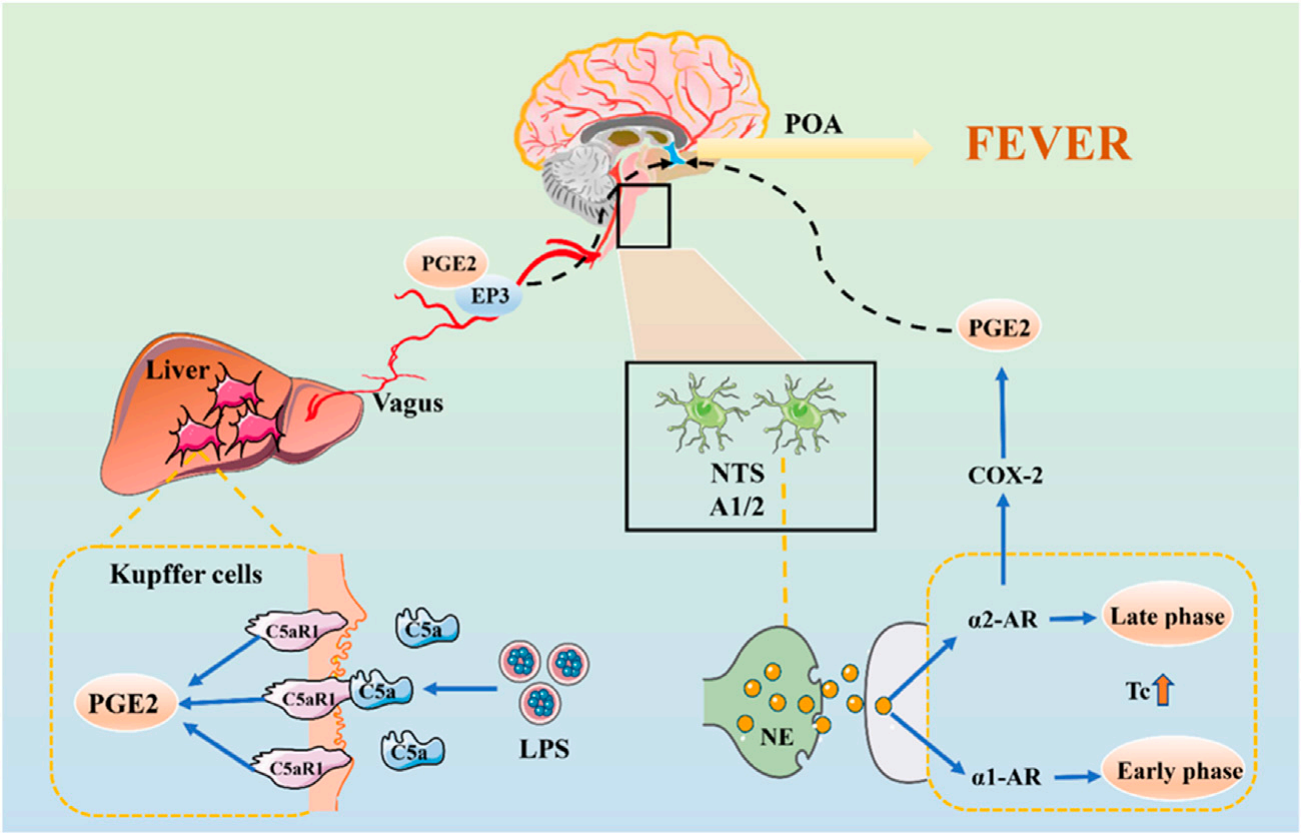
The Neural Transmission Pathway of Fever Signals. The initial stage of fever may be mediated by peripheral PGE2, which is released by KC stimulated by LPS-activated C5a and binding to EP3 receptors. PGE2 is transmitted to the NTS via vagal afferents and is further transmitted via the ventral noradrenergic bundle to the POA, wherein NE is released.

The activation of the neural pathway is believed to be another mechanism for fever (Figure 2). The idea of a neural transmission pathway concerned with fever emerged decades ago. In 1987, Morimoto speculated that peripheral nerves were involved in the development of fever (Morimoto et al., 1987). The messages, utilizing nerve fibers, have the advantages of rapid transmission speed and not being affected by the impedance of the BBB. The rapidity of neural communication between the immune system and the brain seems to be crucial at the beginning of fever (Romanovsky et al., 2000).

Previous studies have shown that bilateral truncal subdiaphragmatic vagotomy or intraperitoneal injection of low doses of capsaicin can desensitize the abdominal sensory nerve and inhibit the fever caused by IV LPS in rats (Romanovsky et al., 1997; Székely et al., 2000). The anterior and posterior branches of the vagus nerve are divided into five main branches under the diaphragm. We have shown that the selective hepatic branch vagotomy plays an important role in the early febrile phase (Simons et al., 1998). The circulating LPS activates KC to produce a factor that stimulates cognate receptors on hepatic vagal afferents, and quickly transmits its fever information to POA (Blatteis, 2006). In the early stage of fever, it may be mediated by peripheral PGE2, which is released by KC stimulated by LPS-activated C5a and binding to EP3 receptors on sensory hepatic vagal afferents.

There may be another mechanism for nerve conduction of fever signals. LPS induce c-fos expression in the nucleus of the solitary tract (NTS) (Wan et al., 1994). PGE2 is produced in the peripheral, introduced into the NTS through vagal afferents and is further transmitted to POA through the ventral noradrenergic bundle, in which norepinephrine (NE) is released (Roth and Blatteis, 2014). The role of the central noradrenergic system in thermoregulation has also been confirmed. NE induces two differentially mediated temperature rises: the first develops promptly and is mediated by the α1-adrenergic receptor (α1-AR), but not by PGE2 (Roth and Blatteis, 2014). The second is mediated by α2-AR and NE induces the production of COX-2/mPGES-1-dependent PGE2 in the POA via an α2-AR-mediated mechanism which is significantly later than the first (Blatteis, 2006). PGE2 also enhances the production of cAMP of hypothalamic cells, which can change the temperature set-point.

**FIGURE 3:**
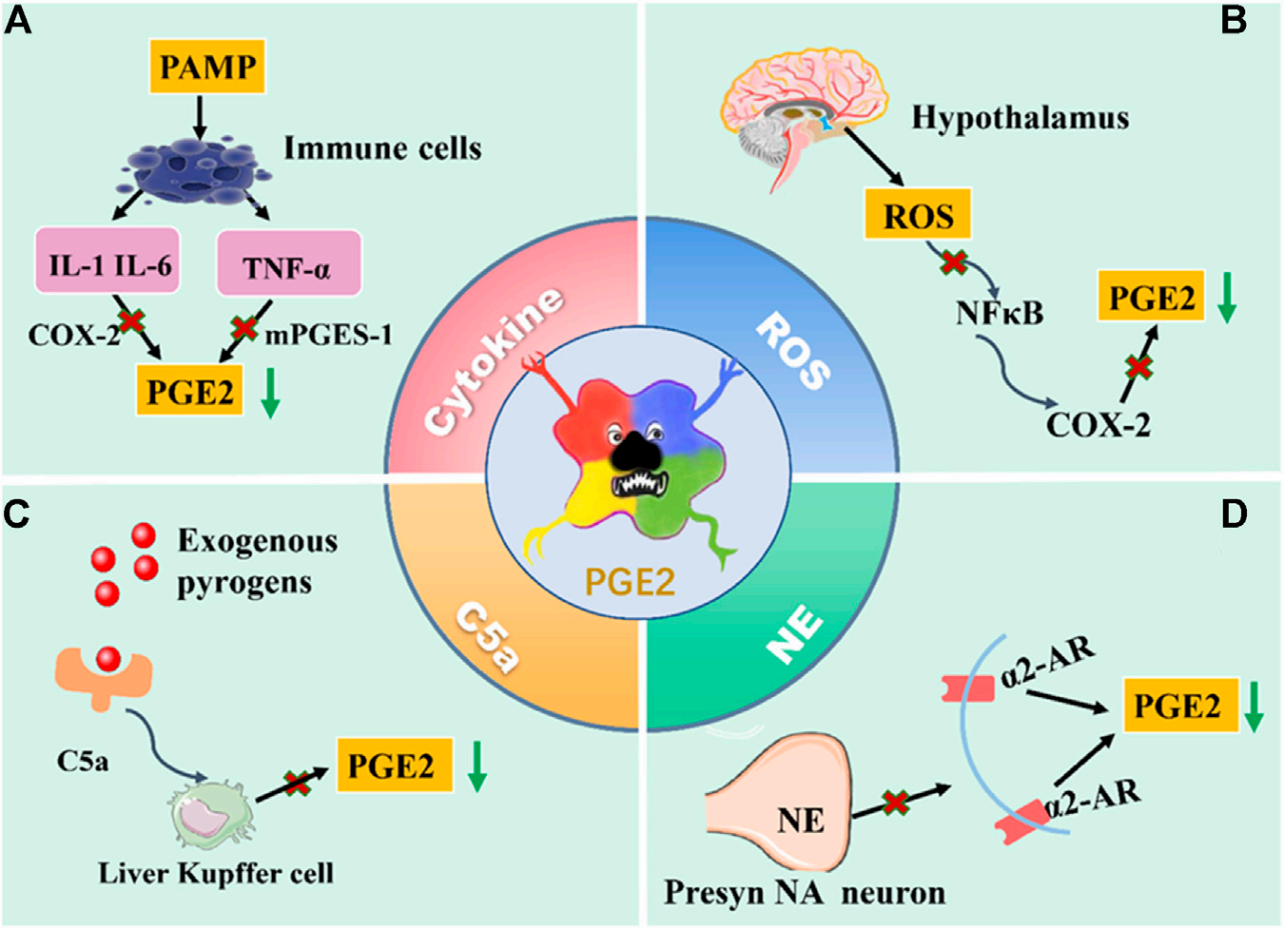
The production way of PGE2. **(A)** PAMPs induces pyrogenic cytokines through pattern recognition receptors such as TLR on immune cells. IL-1and IL-6 can activate COX-2 to produce PGE2; TNF-α activates the mPGES-1 and produces PGE2. **(B)** In the fever state, the overproduction of ROS in the hypothalamus stimulates the activation of NF-κB and expression of COX-2, leading to the generation of PGE2. **(C)** LPS-activated complement can rapidly trigger KC to activate COX-1 to catalyze PGE2 production. **(D)** NE induces the production of COX-2/mPGES-1-dependent PGE2 in the POA via an α2-AR-mediated mechanism. Above, the antipyretic effect was achieved by blocking the production pathway of PGE2 and reducing the content of PGE2.

Generally speaking, fever is the result of complex and periodic interactions between the fever medium and the body. The mechanism of its occurrence is complex. From the material basis of fever and the transmission of fever signals, PGE2 is the vital medium of fever, whether infectious or noninfectious fever. Its means of production are 1) invasion of exogenous pyrogen to the host triggers a series of immune responses through PAMPs. PAMPs induces pyrogenic cytokines (IL-1, IL-6, and TNF- *α*) through pattern recognition receptors such as TLR on immune cells. IL-1 can activate MAPKp38 and c-Jun by TAK1 to induce COX-2 and produce PGE2; it can also induce COX-2 and PGE2 by activating the NF-κB pathway. After binding IL-6 with IL-6R on brain endothelial cells, COX-2 expression can be induced by intracellular signaling of the STAT3 pathway to produce PGE2. TNF-α activates the mPGES-1 and produces PGE2 under the glutathione mediated signal pathway. 2) In the fever state, the overproduction of ROS in the hypothalamus stimulates the activation of NF-κB and expression of COX-2, leading to the generation of PGE2. 3) LPS-activated complement can rapidly trigger KC to activate COX-1 to catalyze PGE2 production. 4) NE induces the production of COX-2/mPGES-1-dependent PGE2 in the POA via an α2-AR-mediated mechanism (as shown in Figure 3).

The humoral pathway transmits the first two kinds of fever signals; the neural pathways mediate the rest. The above understanding of the fever mechanism also broadens a new way of thinking for antipyretic. The antipyretic effect can be achieved by inhibiting the production of PGE2 in various ways and blocking its role in the fever pathway. Recent research data on the fever medium indicates that some endogenous mediums may cause fever. These studies show that in addition to PGE2, the mediators produced by the center also participate in the fever response. At present, the corticotropin-releasing factor, endothelin, and macrophage inflammatory protein one are the most studied central agents of fever. However, whether the antipyretic effect is independent of PGE2 is still controversial. Therefore, the antipyretic mechanism needs further study.

## Single Chinese Herbal Medicines in the Treatment of Fever

### Bupleuri Radix

Bupleuri Radix (BR), called Chaihu in Chinese, is the dried roots of *Bupleurum chinense* DC and *Bupleurum scorzonerifolium* Willd. The compounds of BR include essential oils, triterpenoid saponins, polyacetylenes, flavonoids, lignans, fatty acids, and sterols (Yang et al., 2017). Jin et al. (2014) researched the effect of BR water extract, BR saponin extract, and BR essential oil extract on the fever model of rats via subcutaneous injection of a yeast suspension. It was observed that the three extracts had a good antipyretic effect. Chen et al. (2010) extracted the essential oil from BR to prepare the gel and sprayed it into the nasal cavity of febrile rabbits that had undergone IV injected *Escherichia coli* endotoxin. The results showed that the essential oil of BR could play an antipyretic effect on the rabbit's fever by decreasing the concentration of cAMP in the cerebrospinal fluid. One study demonstrated that intracerebroventricular injection of saikosaponin A (SSA) reduces the core temperature of rats with fever caused by IL-1β after 30 min. At the same time, the content of cAMP in serum and protein kinase A (PKA) in the cytoplasm was significantly lower than those in the model group. The results showed that SSA could be used as an antipyretic by reducing cAMP secretion and PKA activity in the hypothalamus (Sun et al., 2016). The study demonstrated that SSA can significantly inhibit the expression of TNF-α, IL-1β, and IL-6; it also inhibits the activation of the NF-κB signaling pathway by suppressing the phosphorylation of inhibitory IκBα (Zhu et al., 2013). The above studies showed that BR could play an antipyretic role by inhibiting the secretion of cAMP, the expression of pyrogenic cytokines, the activity of PKA, and the activation of the NF-κB signaling pathway.

### Scutellariae Radix

Scutellariae Radix (SR), known as Huangqin in Chinese, is the dried root of the Labiatae plant *Scutellariae baicalensis* Georgi (Bruno et al., 2002). Flavonoids, phenylethanoid glycosides, iridoid glycosides, alkaloids, phytosterols, and polysaccharides are the main compounds of SR (Li et al., 2011). SR has an antipyretic effect in the clinical setting. In recent years, the antipyretic mechanism of SR and its components have been studied intensely. The results show that baicalin and baicalein are main active components of SR for reducing fever (Yang and Meng, 2009). The body temperature of rats with fever-induced by yeast decreased significantly after the administration of baicalin by gavage. The contents of IL-6, IL-1β, and TNF-α in serum, hypothalamus, and CSF of rats decreased correspondingly, indicating that baicalin has an antipyretic effect by reducing the content of pyrogenic cytokines (Li and Ge, 2010). Baicalin was used to treat rats with fever induced by intraperitoneal injection of LPS. The results showed that baicalin significantly reduced the body temperatures of the fever rats. The mechanism may be that baicalin can inhibit the up-regulation of TLR4 mRNA and protein expression regulated by LPS, downregulate NF-κB activation and decrease the expression of TNF-α, IL-1β protein (Ye et al., 2015). Baicalin can inhibit the fever response induced by LPS and the excessive production of glutamate and hydroxyl radicals in the hypothalamus caused by central administration of TNF-α. These results suggest that the antipyretic effect of baicalin may be achieved by inhibiting the MNDA receptor-dependent hydroxyl radical pathways in the hypothalamus and circulating TNF-α accumulation during LPS-induced fever (Tsai et al., 2006). Nakahata et al. (1998) found that Ca2^+^ ionophore A23187 caused phosphorylation of MAPK, resulting in the activation of cytosolic phospholipase A2 (cPLA2), baicalein reduces the A23187 induced PGE2 release by inhibition of AA liberation through the inhibition of the MAPK-cPLA2 pathway. Baicalein inhibit the expression of COX-2 induced by LPS in Raw 264.7 cells, through blockading C/EBPb DNA binding to the COX-2 promoter, thereby inhibiting the expression of COX-2 and the production of PGE2 (Woo et al., 2006). In conclusion, SR can play an antipyretic role by inhibiting the release of pyrogenic cytokines and PGE2, the activation of NF-κB, and the excessive production of hydroxyl radicals.

### Chuanxiong Rhizome

Chuanxiong Rhizome (CX), also known as Chuanxiong in Chinese, the dried rhizome of Conioselinum anthriscoides 'Chuanxiong' (syn. *Ligusticum chuanxiong* Hort) The main effective components of CX are phthalides, terpenes, polysaccharides, alkaloids, and essential oil (Chen et al., 2018). A large number of studies have shown that the CX essential oil has an antipyretic effect. The body temperature of the rats with fever caused by subcutaneous injection of 20% yeast suspension was significantly decreased after CX essential oil administration; the expression of COX-2 in the hypothalamus and the production of PGE2 in the central thermoregulation of rats were inhibited (Yang et al., 2009). It has also been shown that the antipyretic action of CX may reduce the content of cAMP in the hypothalamus of the rats with fever-induced by yeast, to make the temperature set-point decrease and reach an antipyretic effect (Yang et al., 2008). It was found that in rabbits with fever caused by ET, CX not only played an antipyretic role but also change the proportion of hypothalamus 5-HT and NE (Li et al., 2003). It is suggested that the change of monoamine neurotransmitter content in the center is one of the antipyretic mechanisms of CX essential oil. Two phthalide lactones, Z-ligustilide and senkyunolide A, were identified from CX essential oil and characterized as inhibitors of TNF-α production in monocytes by LPS. They can also exhibit significant suppressive effects on TNF-α-mediated NF-κB activation (Ran et al., 2011). In conclusion, the antipyretic effect of CX is related to the inhibition of the expression of COX-2, PGE2, the reduction of cAMP in the hypothalamus, the regulation of the neurotransmitter, and suppressive effects on TNF-α-mediated NF-κB activation.

### Cinnamomi Ramulus

Cinnamomi Ramulus (CR), also called Guizhi in Chinese, is the dried twigs of *Cinnamomum cassia* (L.) Presl. It consists of phenylpropanoids, terpenoids, aliphatics, and its glycosides, sterols, flavonoids, and organic acids. Phenylpropanoids such as cinnamaldehyde have been considered the characteristic constituents of CR (Liu et al., 2019a). Cinnamaldehyde has been proved to have an antipyretic effect. An *in vitro* study showed that the expression of transient receptor potential vanilloid 1 (TRPV1) mRNA in the primary dorsal respiratory group (DRG) neurons was significantly upregulated at both 37°C and 39°C after incubation with different concentrations of cinnamaldehyde. The findings might explain the part of the mechanisms of the antipyretic action of cinnamaldehyde, which is achieved through a non-TRPA1 channel pathway (Sui et al., 2010). Rat cerebral microvascular endothelial cells (RCMEC) were cultured in M199 medium containing IL-1β in the presence or absence of cinnamaldehyde. The results showed that cinnamaldehyde inhibited IL-1β-induced PGE2 production through the inhibition of COX-2 activity in cultured RCMEC (Guo et al., 2006). It provides some pharmacological evidence for clinical use of CR in fever. By using a yeast-induced fever model and IL-1β stimulated rat brain microvascular endothelial cells (bEnd.3) as an experimental system to determine the content of PGE2 in the hypothalamus and the supernatant of bEnd.3 (Ma et al., 2008). The results showed that cinnamaldehyde could effectively inhibit the fever response induced by yeast in rats, significantly reduce the content of PGE2 in the hypothalamus of rats with fever, and also inhibit the release of PGE2 stimulated by IL-1. The above studies show that CR can not only relieve fever through the non-TRPA1 channel but also reduce the activity of COX-2 in brain endothelial cells and the content of PGE2 in the hypothalamus of febrile rats.

### Forsythiae Fructus

Forsythiae Fructus (FF), also called Lianqiao in Chinese, is the fruit of *Forsythia suspensa* (Thunb.) Vahl. Currently, compounds have been found in FF, including lignans, phenylethanoid glycosides, flavonoids, terpenoids, cyclohexyl-ethanol derivatives, alkaloids, steroidals, and other compounds (Wang et al., 2018b). According to the clinical practice in TCM, FF has a significant antipyretic effect. Essential oil and forsythoside a (FTA) have been demonstrated to have an antipyretic effect (Dang et al., 2017). Subcutaneous injection of yeast caused fever in rats. The body temperature and the content of cAMP and PGE2 in the hypothalamus of the rats were observed after 2 h. The results showed that both the essential oil and the extract of FF significantly reduced the body temperature of rats with fever. The extract of FF can downregulate cAMP and PGE2 in the hypothalamus. The FF essential oil has an antipyretic effect by downregulating cAMP in the hypothalamus (Dang et al., 2017). Previous studies have suggested that FTA can reduce the temperature of the yeast-induced fever mice. FTA significantly downregulated the expression of TRPV1 in hypothalamus and DRG of the yeast-induced fever mice, inhibited MAPKs activation of the hypothalamus and DRG, and then decreased secretion ofPGE2 (Liu et al., 2017a). Recent studies have shown that forsythoside can significantly reduce TNF-α secretion in LPS-stimulated RAW 264.7 cells, suggesting that it can reduce TNF-α secretion to relieve fever (Guan et al., 2013). FTA can also suppress LPS-mediated induction of the TLR4 pathway. LPS combined with TLR4 activated NF-κB through the primary response gene-88 (MyD88)-independent pathways. Therefore, FTA may inhibit TNF-α and NF-κB by blocking LPS/TLR4 signaling pathway (Zeng et al., 2017). Generally, FF may play an antipyretic role by reducing cAMP, PGE2, and TNF-α in the hypothalamus, suppressing TRPV1 expression, and the LPS/TLR4 signaling pathway.

### Lonicera Japonica Flos

Lonicera Japonica Flos (LJ), also known as Jin Yin Hua, is the dry flower buds of *Lonicera japonica* Thunb. It is abundant with iridoids, essential oil, flavones, organic acids, and triterpenoid saponins (Shang et al., 2011). The aqueous extract from LJ has been used in TCM for treating fever for thousands of years (Xu et al., 2007). In rabbits with fever caused by IL-1β, based on observing the antipyretic effect of LJ, the expression of prostaglandin receptor EP3 in the preoptic-anterior hypothalamus (POAH) of New Zealand rabbits was detected. The results showed that LJ has an antipyretic effect, and its mechanism may be related to the inhibition of EP3 expression in POAH (Xie et al., 2009). The current study investigated the effect of LJ water extracts on inhibition of both COX-1 and COX-2 activity. The result showed that the inhibitory effect of LJ water extract on COX-2 activity after boiling was four times. The COX-2 transcriptional inhibition of boiled LJ extracts may be due to the action of bifidoflavonoids or similar compounds through NF-κB (Xu et al., 2007). The antipyretic effect of LJ may inhibit the synthesis of PGE2 by inhibiting the activity of COX-2, as well as inhibiting EP3 expression in POAH. Chlorogenic acid (CGA) is the main active component of LJ, however, CGA did not inhibit LPS induced fever even at the highest test dose (200 mg/kg) (Dos Santos et al., 2006). Therefore, CGA may lack antipyretic activity; the material basis of the antipyretic effect of LJ needs further study.

### Other Single Chinese Herbal Medicines of Antipyretics

**FIGURE 4:**
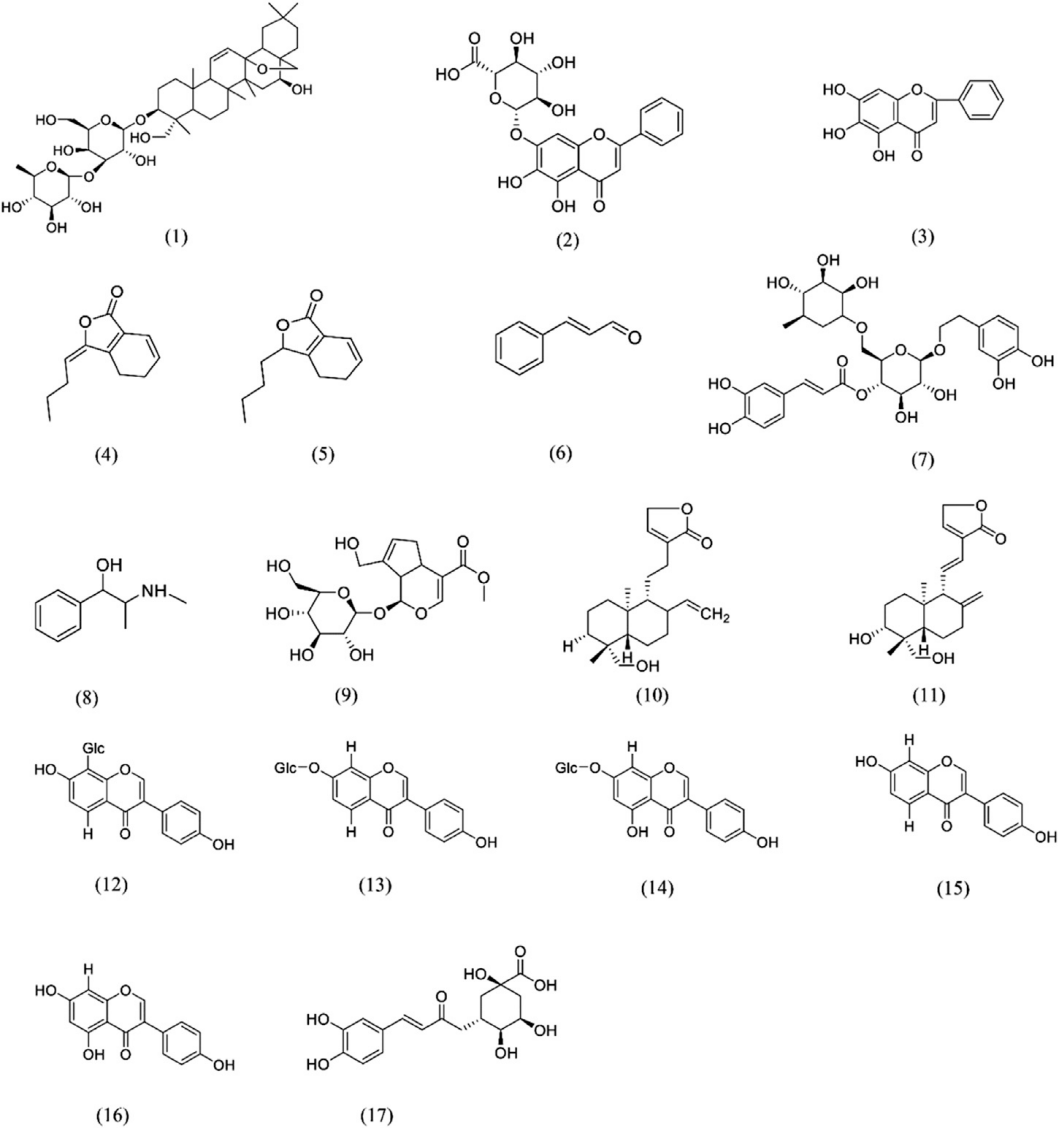
Chemical structures of e phytochemicals that possess antipyretic activity (1)Saikosaponin A; (2) Baicalin; (3) Baicalein; (4) Z-ligustilide; (5) Senkyunolide A; (6) Cinnamaldehyde; (7) Forsythoside A; (8) Ephedrine; (9) Geniposide; (10) Andrographolide; (11) 14-deoxy-11,12-didehydroandrographolide; (12) Puerarin; (13) Daidzin; (14) Genistin; (15) Daidzein; (16) Genistein; (17) Chlorogenic acid.

The above are six commonly used TCM in clinical practice. Their active components play an antipyretic role through different mechanisms. The chemical structure formula is shown in Figure 4. In addition to the above six commonly used TCM (Table 2), Ephedrae Herba, Gypsum Fibrosum, Gardeniae Fructus, Bubali Cornu, Houttuyniae Herba, Andrographis Herba, and Puerariae Lobatae Radix also have significant antipyretic effects (Table 3).

**TABLE 2:** The single Chinese herbal medicine of antipyretic.

Chinese herbal medicine	Scientific name	Bioactive components	Mechanisms
Bupleuri radix	*Bupleurum chinense* DC and *Bupleurum scorzonerifolium* Willd	Essential oilsaikosaponin a (1)	Decrease the concentration of cAMP; Reduce cAMP secretion and PKA activity in the hypothalamus; Inhibit the expression of TNF-α, IL-1β, IL-6 and the activation of the NF-κB signaling pathway
Scutellariae radix	*Scutellaria baicalensis* Georgi	Baicalin (2)Baicalein (3)	Inhibit the upregulation of TLR4 mRNA; Downregulated NF-κB activation with simultaneous decreases in TNF-α and IL-1β protein expression; Suppress glutamate and hydroxyl radicals in the hypothalamus; Inhibit the expression of COX-2 and PGE2
Chuanxiong rhizome	Conioselinum anthriscoides ‘Chuanxiong’ (syn. *Ligusticum chuanxiong* Hort)	Z-ligustilide (4) senkyunolide a (5)	Inhibit the expression of COX-2 and PGE2; Reduce the content of cAMP in the hypothalamus; Change of monoamine neurotransmitter content in the center; Suppressive effects on TNF-α-mediated NF-κB activation
Cinnamomi ramulus	*Cinnamomum cassia* (L.) J.Presl	Cinnamaldehyde (6)	Up-regulated the expression of TRPV1 in DRG neurons; Reduce the activity of COX-2 in brain endothelial cells and the content of PGE2 in the hypothalamus of febrile rats
Forsythiae Fructus	*Forsythia suspensa* (Thunb.) Vahl	Essential oilForsythoside a (7)	Reduce the content of cAMP in the hypothalamus; Suppress TRPV1 expression and activation, inhibiting MAPKs activation of the hypothalamus and DRG
			Inhibit TNF-α and NF-κB through blockade of the LPS/TLR4 signaling pathways
Lonicera japonica Flos	*Lonicera japonica* Thunb	—	Inhibit the synthesis of PGE2 by inhibiting the activity of COX-2; Inhibit EP3 expression in POAH

**TABLE 3:** The other single Chinese herbal medicine of antipyretic.

Chinese herbal medicine	Scientific name	Bioactive components	Model	Drug delivery cycle	Mechanisms	The species investigated	References
Ephedrae Herba	*Ephedra sinica* Stapf, *Ephedra intermedia* Schrenk et C. A. Mey and *Ephedra equisetina* Bunge.	Ephedrine (8)	Subcutaneous injection of 20% yeast water suspension (10 ml/kg) to induce fever	Ephedrae Herba extracts were administered 8.1 g/kg	Reduce the level of 5-hydroxytryptamine and NE in the hypothalamus	Fifty Wistar rats (weighing 200 ± 20 g)	Wang et al. (2018a)
Gypsum Fibrosum	—	—	Subcutaneous injection of 15% yeast suspension (10 ml/kg) to induce fever	Intragastric administration of Gypsum suspension for 7 days (10 g/kg)	Reduce the synthesis of PGE2	Aged SD rats (weighing200–250 g)	TangZhishu and Bing (2012)
Gardeniae Fructus	*Gardenia jasminoides* J.Ellis	Geniposide (9)	15% saline suspension of yeast was injected in the back of rats (10 ml/kg)	Administered with Gardeniae Fructus at 4.5 g/kg (10 ml/kg)	Reduce the expression of IL-6 and TNF-α; Reduce the production of PGE2	Male SD rats (weighing 170 ± 10 g)	Zhang X. et al. (2019)
Bubali Cornu	*Bubalus bubalis* Linnaeus	—	Fever caused by subcutaneous injection of 20% yeast (10 ml/kg)	400 mg/kg Bubali Cornu powder extract was administrated orally with a dosage of 10 ml/kg	Change the metabolism of uric acid and cysteine; enhance the activity of antioxidant enzymes; reduce the level of TNF - α; reduce the ROS production and PGE2 synthesis	Aged SD rats (weighing 200 ± 20 g)	Liu et al. (2016)
Houttuyniae Herba	*Houttuynia cordata* Thunb	—	Fever caused by subcutaneous injection of 15% yeast suspension (10 ml/kg)	Three hours after the establishment of the model, 20, 10, and 5 ml/kg were administrated intravenously in the high, middle and low dose groups	Inhibit the production of 1L-1, TNF-α, and the expression of PGE2	Aged male SD rats	Zhang et al. (2010)
Andrographis Herba	*Andrographis paniculata* (Burm. f.) Nees	Andrographolide (10)14-deoxy11,12-didehydroandrographolide (11)	Rats were injected subcutaneously with yeast (0.135 g/kg)	Two hours after yeast injection,4 mg/kg of andrographis Herba extracts were injectedIntraperitoneally	Inhibit the expression of NF-kB, reducing the expression COX-2 and the level of PGE2	SD rats	Suebsasana et al. (2009)
Puerariae Lobatae radix	*Pueraria montana var. lobata* (Willd.) Maesen & S.M.Almeida ex Sanjappa & Predeep (syn. *Pueraria lobata* (Willd.) Ohw)	Puerarin (12) daidzin (13)Daidzein (14)Genistin (15)Genistein (16)	Fever caused by subcutaneous injection of LPS (50 mg/kg)	The experimental groups received 50 and 100 mg/kg of pueraria extract	Inhibit cyclooxygenase; inhibit PGE2 release From mouse peritoneal macrophages in vitro	Male mice (weighing 22–26 g)	Yasuda et al. (2005)

## Chinese Patent Medicines and Complex Prescriptions in the Treatment of Fever

### Qingkailing Injection

Qingkailing injection (QKLI) is a composite formula of TCM with a significant antipyretic action (Gao et al., 2013a). It comprises eight TCMs or extracts thereof, including Isatidis Radix, Lonicera Japonica Flos, Gardeniae Fructus, Bubali Cornu, Margaritifera Concha, Baicalinum, Acidum Cholicum, and Acidum Hyodesoxy-cholicum (Yan et al., 2005). Plasma pharmacokinetics study of QKLI demonstrated that baicalin 2) and geniposide 9) might be the potential active antipyretic components of QKLI (Zhang et al., 2016). Baicalin and geniposide could be detected in the hypothalamic dialysate after IV administration of QKLI. However, metabolomics biomarkers of QKLI were highly correlated with baicalin while gardenoside has no statistically significant correlation with these biomarkers (Zhang et al., 2017). Therefore, baicalin may be the more important active component for QKLI to play the role of antipyretic. The network pharmacology study of the antipyretic effect of baicalin shows that baicalin can regulate fever-related molecules NO by targeting on caspase 3 (CASP3) and regulate cAMP, PGE2 to produce antipyretic effect (Zhang et al., 2017). The mechanism of antipyretic action of SR includes baicalin; it plays an antipyretic role by inhibiting the release of IL-6, IL-1β, TNF-α, and PGE2, the activation of NF-κB, and the excessive production of hydroxyl radicals. From QKLI, baicalin was the only antipyretic component screened; it alone cannot represent the antipyretic activity of QKLI. Other antipyretic components in QKLI that play their antipyretic role through humoral pathway. In the study of urine metabolomics, it was found that QKLI can reduce the content of tryptophan by 5-HT and repair the disorder of amino acid metabolism on yeast induced fever rats (Gao et al., 2013a). A plasma study shows that QKLI can correct the interference of amino acid metabolism and lipid metabolism to relieve fever (Qin et al., 2016). However, except baicalin, other antipyretic components of QKLI are seldom studied, and its antipyretic components need to be further studied.

### Shuang-Huang-Lian Preparation

SHL is a famous TCM recipe, which was included in Chinese pharmacopoeia in 2015. It contains LJ, SR, and FF; it is clinically used to treat fever and infectious diseases such as acute upper respiratory tract infection (Gao et al., 2014a). SHL products are administered in a variety of different routes (e.g., oral, injectable, and pulmonary routes) (Gao et al., 2014c). Three effective ingredients, chlorogenic acid (17), baicalin (2), and forsythin (7), are officially recorded as quality control standards. Baicalin has an antipyretic effect by inhibiting the release of IL-6, IL-1β, TNF-α, and PGE2, the activation of NF-κB, and the excessive production of hydroxyl radicals (See 6.2). Forsythin may have an antipyretic effect by reducing cAMP, PGE2, and TNF-α in the hypothalamus, suppressing TRPV1 expression, and the LPS/TLR4 signaling pathway (See 6.6). The study first evaluated the antipyretic effect of SHL injection (SHLI) using UPLC-Q-TOF/MS-based metabolomics study to reveal the antipyretic mechanism of SHLI on the yeast-induced pyrexia rat model. The result shows that SHLI might contribute to the repair of lipid metabolism, amino acid metabolism, and energy metabolism to work antipyretic effects (Gao et al., 2014b). It reported that baicalin in SHL was metabolized to baicalein in a study, baicalin was used as a representative compound to study the pharmacokinetics of SHL. It was found that the compound prescription might prolong the effect of baicalin *in vivo* (Di et al., 2006). It suggests that the antipyretic effect of SHL may be better than that of SR. Another study was conducted to compare the antipyretic effect of LJ and SHL on rectal temperature changes induced by yeast. The results indicated that SHL showed a better antipyretic effect than LJ (Gao et al., 2014). In conclusion, the antipyretic effect of SHL is better than that of single herb.

### Others

In addition to the QKLI and SHL series preparations, Jinxin oral liquid, Yin Qiao San, Hao Jia Xu Re Qing Granules, and Reduning injection are used in clinical antipyretic treatment. Gegen Qinlian decoction and Bai-Hu decoction are two classical antipyretic prescriptions that have also been proved to have significant antipyretic effects (Table 4).

**TABLE 4:** Chinese Patent Medicines and Complex Prescriptions of antipyretic.

Chinese patent medicine and complex prescriptions	Components	Scientific name	Model	Drug delivery cycle	Mechanisms	The species investigated	References
Jinxin oral liquid (JXOL)	Ephedra sinica, Descurain Semen, Mori Cortex, Armeniacae Semen Amarum, Gypsum Ustum, Peucedani Radix, Scutellariae Radix, Polygoni Cuspidati Rhizoma et Radix	*Ephedra sinica *Stapf*, Descurainia sophia *(L.)Webb ex Prantl*, Morus alba *L*., Prunus armeniaca *L*., *Gypsum Ustum*, Scutellaria baicalensis Georgi, Reynoutria japonica* Houtt	Fever caused by subcutaneous injection of 20% yeast (15 ml/kg)	Subcutaneous injection of 7.02 g/kg JXOL	Reduce the production of IL-1 β, PGE2 and the level of quinolinic acid and pantothenic acid; regulate the metabolism level of 3-phosphoglycerate, pyruvate and other metabolites	Male SD rats (weighing 80 ± 20 g)	Qian et al. (2019)
Yin Qiao San (YQS)	Lonicera Japonica Flos, Lophatheri Herba, Forsythiae Fructus, Platycodonis Radix, Sojae Semen Praeparatum, Arctii Fructus, Menthae Haplocalycis Herba, Schizonepetae Herba, Phyllostachys Rhizoma, Glycyrrhizae Radix et Rhizoma	*Lonicera japonica *Thunb*, Lophatherum gracile* Brongn, *Forsythia suspensa* (Thunb.) Vahl, *Platycodon grandiflorus* (Jacq.) A.DC., *Glycine max* (L.) Merr., *Arctium lappa* L., *Mentha canadensis* L., *Nepeta tenuifolia* Benth., *Phragmites australis* subsp. Australis, *Glycyrrhiza uralensis* Fisch. ex DC.	Subcutaneous injection of 20% yeast (20 ml/kg) to induce fever	Different doses of YQS solution were respectively gavage administration in fever rats	Reduce the cAMP level of the hypothalamus	Male SD rats (weighing 200 ± 20 g)	Huang and Chang (2016)
Hao Jia Xu Re Qing Granules (HJ)	Artemisiae Annuae Herba, Glycyrrhizae Radix et Rhizoma Trionycis Carapax, Rehmanniae Radix, Dendrobii caulis, Anemarrhenae rhizoma, Moutan cortex, Puerariae Lobatae Radix,	*Artemisia annua* L., *Glycyrrhiza uralensis* Fisch. ex DC., *Trionyx sinensis* Wiegmann, *Rehmannia glutinosa* (Gaertn.) DC., *Dendrobium nobile* Lindl., *Anemarrhena asphodeloides* Bunge, *Paeonia suffruticosa* Andrews, *Pueraria montana var. lobata* (Willd.) Maesen & S.M.Almeida ex Sanjappa & Predeep	Rats were subcutaneously injected with 10 mg/kg of 10% yeast suspension	1.44 g/kg, 0.72 g/kg, 0.36 g/kg of HJ were given by gavage after being injected with yeast	Inhibit the tryptophan metabolism;Reduce the level of 5-HT	SD rats (weighing 200 ± 20)	Yu et al. (2018)
Reduning injection (RDN)	Artemisiae Annuae Herba, Lonicera Japonica Flos, Gardeniae Fructus	*Artemisia annua* L., *Lonicera japonica* Thunb, *Gardenia jasminoides* J.Ellis	Rats were subcutaneously injected with 5 ml/kg of 20% yeast suspension	Rats were I.V.with 6 ml/kg RDN	Reduce the level of IL-1β, IL-6, PGE 2, TNF-α and cAMP in febrile rats;Change the regulation of amino acid metabolism, lipid metabolism and energy metabolism	Male SD rats (weighing180-220 g)	Gao et al. (2020)
Gegen Qinlian decoction (GQLD)	Puerariae lobatae Radix, Scutellariae Radix, Coptidis Rhizoma, Glycyrrhizae Radix et Rhizoma Praeparata Cum Melle	*Pueraria montana var. lobata* (Willd.) Maesen & S.M.Almeida ex Sanjappa & Predeep, *Scutellaria baicalensis* Georgi, *Coptis teeta* Wall., *Glycyrrhiza glabra* L.,	Rats were subcutaneously injected with 10 ml/kg of 20% yeast suspension	Rats were orally administered with GQLD (1.728 g/kg)	Regulate the metabolisms of phospholipid, sphingolipid, fatty acid oxidation, fatty acid amides, amino acid and glycerolipid in vivo	Male Wistar rats weighing (240 ± 20 g)	Liu et al. (2019b)
Bai-Hu decoction (BHD)	Gypsum Ustum, Anemarrhena Rhizoma, Glycyrrhizae Radix et Rhizoma Praeparata Cum Melle, Rice	Gypsum Ustum, *Anemarrhena asphodeloides* Bunge, *Glycyrrhiza glabra* L., Rice	Intravenous injection of LPS (200 ng/kg)	Orally administered with BHD 7 ml/kg	Reduce the content of IL-1β and TNF-α in serum, and TNF-α in hypothalamus	New Zealand rabbits (weighing 2.0–3.0 kg)	Jia et al. (2013)

## Clinical Trials

In the study of the antipyretic mechanism of TCM, various models have been developed to simulate the natural fever of experimental animals. However, fever is an important clinical manifestation of many diseases, so most of the clinical trials of antipyretics in TCM are focused on acute upper respiratory tract infection, acute tonsillitis, acute otitis media, and other diseases (Li, 2002; Zhang et al., 2013b). At present, there are a few clinical trials on the antipyretic effect of single Chinese herbal medicines (Table 5). The clinical trials of antipyretic Chinese patent medicine mainly include SHL series preparations and QKLI. It's worth noting that the clinical trials of antipyretic TCMs are concentrated on infectious fever, but high-quality clinical trials are lacking. Clinical trials study to evaluate the effect of antipyretic TCMs will use more strict protocols, concealment of allocation, and double-blinding, in order to ensure the compliance of international acceptable standards.

**TABLE 5:** Clinical trials of Chinese Patent Medicines of antipyretic.

Chinese patent medicines	Disease	Subject	Study design	Intervention	Length	Outcome		References
Shuang-Huang-Lian injection	Acute tonsillitis	90 subjects (56 men and 64 Women)	Randomized controlled trials	1 ml/kg·d SHLI diluted with 5% glucose injection 500 ml or physiological saline 500 ml, intravenous drip, once a day	5–7 days	Fever resolution		Yan et al. (2007)
Shuang-Huang-Lian lyophilized powder for injection	Acute upper respiratory tract infection	98 subjects (43 men and 55 Women)	Randomized controlled trials	3 g Shuang-Huang-Lian lyophilized powder diluted with physiological saline 500 ml, intravenous drip, once a day	4–7 days	Fever resolution	Ouyang et al. (2008)		
Shuang-Huang-Lian oral liquid	Acute tonsillitis	72 children (36 male and 36 Female)	Randomized controlled trials	One for children aged 1–3, three times a day; two for children aged 4–7, three times a day	7 days	Fever resolution Reduce IL-6 and TNF-α in serum	Ye (2019)		
Shuang-Huang-Lian injection (SHLI)	Acute tonsillitis	120subjects (61 men and 59 Women)	Randomized controlled trials	20 ml SHLI was diluted in 20 ml physiological saline for ultrasonic atomization inhalation treatment, twice a day	14 days	Fever resolution;Reduce IL-6 and TNF-α in serum	Fu et al. (2019)		
Shuang-Huang-Lian lyophilized powder for injection	Acute tonsillitis	46 subjects (20 men and 26 Women)	Randomized controlled trials	3 g Shuang-Huang-Lian lyophilized powder diluted with physiological saline 500 ml, intravenous drip, once a day	5–7 days	Fever resolution	Jiang and Wang (2019)		
Shuang-Huang-Lian oral liquid	Bacterial respiratory infection	46 subjects of both sexes	Randomized controlled trials	20 ml Shuang-Huang-Lian oral liquid, three times a day	3–5 days	Fever resolution	Yang (2018)		
Qingkailing injection (QKLI)	Acute upper respiratory tract infection	46 subjects (24 men and 21 Women)	Randomized controlled trials	20 ml QKLI diluted with 5% physiological saline 250 ml, intravenous drip, once a day	7 days	Fever resolution	Fan and Liu (2011)		
Qingkailing injection	Acute upper respiratory tract infection	80subjects (43 men and 37 Women)	Randomized controlled trials	20 ml QKLI diluted with 5% physiological saline 250 ml, intravenous drip, once a day	7 days	Fever resolution	Zhang (2019)		
Qingkailing injection	Febrile convulsion in children	50 subjects of both sexes	Randomized controlled trials	15 ml for 3-4-year-old children and 30 ml for 5-6-year-old children, it was added into 10% glucose injection for intravenous drip once a day	4 days	Fever resolution; Reduce IL-β, cAMP and TNF-α	Yan and Fan (2019)		
Qingkailing injection	Acute upper respiratory tract infection with high fever	54 subjects of both sexes	Randomized controlled trials	16–40 ml QKLI diluted with 5% glucose injection 250 ml or physiological saline 250 ml, intravenous drip once a day	3–7 days	Fever resolution	Xian et al. (2010)		
ReduningInjection (RDNI)	Fever, rash, and ulcers in children	120 subjects of both sexes	Randomized, double-blind, parallel controlled, and multicenter clinical trial	Patients 1–5 years old,RDNI was given at 0.5 ml/kg per day with a maximal dosage of 10 ml; patients 6–10 years old, 10 ml RDNI was given; patients 11–13 years old, 15 ml RDNI was given once a day	3–7 days	Reduction in onset time of antifebrileEffect, an acceleration of body temperatureRecovery, and a stability of body temperature after fever reduction	Zhang et al. (2013a)		
ReduningInjection (RDNI)	Acute upper respiratory tract infection with fever	123 subjects of both sexes	Randomized controlled trials	0.6 ml/kg RDNI diluted with physiologicalSaline100ml, intravenous drip once a day	3 days	Fever resolution	Li (2013)		
Yin Qiao San (YQS)	Acute upper respiratory tract infection with fever	327 subjects of both sexes	Randomized, double blind placebo-controlled trial	Two 7 g sachets, twice a day	10 days	Fever resolution	Wong et al. (2012)		
Yin Qiao San	Viral influenza	124 subjects of both sexes	Randomized, single blind clinical trial	The herbs decoct until about 300 ml. A daily dose, with warm water, twice a day	5 days	Fever resolution	Huang et al. (2013)		

## Conclusion and Perspectives

Since the global outbreak of the infectious disease COVID-19 in 2019, China has taken strong measures to quickly engage in the fight against the COVID-19. TCM has played an important role in the prevention and treatment of COVID-19 because of its unique insights and experiences. For patients with mild symptoms, TCM early intervention can effectively prevent the disease from turning into severe. In severe cases, TCM improves the symptoms to win the rescue time for the patients. TCM has own characteristics such as holistic concept, syndrome differentiation and treatment, strengthening the body resistance to eliminate pathogenic factors. Judging from the current treatment plan, TCM treats patients with COVID-19 based on the idea of syndrome differentiation and treatment. In order to improve the fever in some patients, prescriptions need to be compatible with antipyretic TCMs.Therefore, this paper summarized the mechanisms of fever from two aspects of pathology and physiology. On this basis, combined with the chemical composition and pharmacological action of TCM, it analyzed the mechanisms of the antipyretic effects of TCM through various ways, so as to provide reference for the efficient utilization of existing drugs.

It must be pointed out that in the face of the spread of new epidemics, due to the particularity of the disease and the urgency of formulating effective diagnosis and treatment plans, suitable treatment models have been developing based on basic theories and clinical experiences. Therefore, it is important to choose drugs with a clear therapeutic effect and clinical basis. The single Chinese medicine and compound preparations of TCM which have specific antipyretic effects listed in this paper are widely used in clinical practice, and can effectively improve the symptoms of fever in acute respiratory infections and control infections. TCM has the unique properties of multi-components and multi-targets. The majority of these mentioned drugs may not only exert the effects of antipyretic, but also have the properties of anti-inflammation, and immunity enhancement; and some of them are antiviral, and may be promising medicines for the treatment or adjuvant treatment of COVID-19 patients.

## Glossary

TCM: traditional chinese medicine
NSAIDs: nonsteroidal anti-inflammatory drugs
Poly-IC: polyinosinic polycytidylic acid
ACE-2: angiotensin-converting enzyme-2
LPS: lipopolysaccharides
PAMPs: pathogen-associated molecular patterns
TLRs: toll-like receptors
IL-1: interleukin-1
IL-6: interleukin-6
TNF-α: tumor necrosis factor α
IL-1RA: IL-1 receptor antagonist
PGE2: prostaglandin E2
Cox-2: cyclooxygenase 2
IKK2: IκB kinase 2
rh TNF: human recombinant TNF
mPGES-1: PGE synthase-1
I.V: intravenous
I.C.V: intra cerebro-ventricular
BBB: blood-brain barrier
Kc: kupffer cells
C5a: complement component 5a
PLA2: phospholipase A2;
IL-6R: IL-6 receptor
NMDA: N-methyl-D-aspartate
ROS: reactive oxygen species
iNOS: inducible nitric oxide synthetase
5-HT: 5-hydroxy tryptamine
GABA: γ-aminobutyric acid
OVLT: organum vasculosum of lamina terminalis
POA: preoptic-anterior hypothalamic area
AA: arachidonic acid
CVOs: circumventricular organs
NTS: nucleus of the solitary tract
NE: norepinephrine
PKA: protein kinase A
IκBα: NF-κB inhibitor α
RCMEC: rat cerebral microvascular endothelial cells
bEnd.3: brain microvascular endothelial cells
FTA: forsythoside a
DRG: dorsal respiratory group
POAH: preoptic anterior hypothalamus
CGA: Chlorogenic acid
AURI: acute upper respiratory tract infection
